# The Gut–Muscle Axis in Sarcopenia: Mechanisms, Evidence Gaps and Translational Challenges

**DOI:** 10.3390/biomedicines14050976

**Published:** 2026-04-23

**Authors:** Stefano Andrea Marchitto, Gabriele Abbatecola, Rola S. Zeidan, Lauren Morgan, Riccardo Calvani, Anna Picca, Mathias Schlögl, Matteo Tosato, Christiaan Leeuwenburgh, Stephen D. Anton, Francesco Landi, Emanuele Marzetti, Stefano Cacciatore

**Affiliations:** 1 Gastroenterology Unit, Department of Clinical Medicine and Surgery, University of Naples Federico II, 80131 Naples, Italy; stefanoandreamarchitto@gmail.com; 2 Department of Geriatrics, Orthopedics and Rheumatology, Università Cattolica del Sacro Cuore, 00168 Rome, Italy; gabrieleabbatecola@gmail.com (G.A.); riccardo.calvani@unicatt.it (R.C.); matteo.tosato@policlinicogemelli.it (M.T.); francesco.landi@unicatt.it (F.L.);; 3 Department of Physiology and Aging, College of Medicine, University of Florida, Gainesville, FL 32611, USA; rzeidan@ufl.edu (R.S.Z.); laurenmorgan@ufl.edu (L.M.); santon@ufl.edu (S.D.A.); 4 Department of Health Outcomes and Biomedical Informatics, College of Medicine, University of Florida, Gainesville, FL 32611, USA; 5 Fondazione Policlinico Universitario “Agostino Gemelli” IRCCS, 00168 Rome, Italy; 6 Department of Medicine and Surgery, LUM University, 70010 Casamassima, Italy; picca@lum.it; 7 Division of Geriatric Medicine, Clinic Barmelweid, 5017 Erlinsbach, Switzerland; mathias.schloegl@barmelweid.ch; 8 Department of Medicine, Division of Geriatric Medicine, College of Medicine, University of Florida, Gainesville, FL 32611, USA; cleeuwen@ufl.edu

**Keywords:** sarcopenia, aging, gut–muscle axis, gut microbiota, dysbiosis, short-chain fatty acids, inflammation, mitochondrial dysfunction, physical exercise, microbiota-targeted interventions

## Abstract

Sarcopenia is an age-related skeletal muscle disorder characterized by reduced muscle mass, strength, and physical performance, as well as increased risk of disability, hospitalization, and mortality. Emerging evidence suggests that gut microbiota alterations may contribute to muscle decline via a microbiota–gut–muscle axis, acting as a context-dependent modulator rather than a primary causal driver. This narrative review synthesizes mechanistic, clinical, and translational evidence linking gut dysbiosis to sarcopenia. Preclinical studies show that microbiota modulation (e.g., antibiotics, probiotics, prebiotics, postbiotics, fecal microbiota transplantation) affects muscle mass, strength, and metabolism through pathways including inflammation, mitochondrial dysfunction, altered short-chain fatty acid production, and impaired anabolic signaling. In humans, observational studies associate lower microbial diversity and reduced short-chain fatty acid-producing taxa with poorer muscle outcomes, but findings are heterogeneous and non-causal. Interventional trials remain limited and characterized by small sample sizes, with effects more consistent for functional outcomes than muscle mass. Overall, the gut microbiota represents a modifiable contributor within the complex biology of sarcopenia. Future studies should integrate microbiome profiling and multi-omics approaches within well-designed clinical trials to identify responder phenotypes and define the role of microbiota-targeted strategies within multimodal interventions.

## 1. Introduction

Sarcopenia represents the pathological manifestation of the progressive decline in skeletal muscle mass and strength occurring with aging, and is associated with adverse clinical outcomes, including falls, disability, hospitalization, and mortality [1,2,3]. Although its pathophysiology is multifactorial, involving anabolic resistance, chronic low-grade inflammation, and metabolic dysfunction, the mechanisms underlying age-related muscle loss remain only partially understood [4].

In parallel with musculoskeletal aging, the gut microbiota undergoes significant compositional and functional changes across the lifespan [5]. Age-related alterations in gut microbiota, often referred to as dysbiosis, are characterized by reduced microbial diversity, increased inter-individual variability, and expansion of potentially pro-inflammatory taxa [6]. These changes have been identified in several age-associated conditions, including metabolic disorders, immune dysfunction, and chronic inflammation [7].

Growing evidence suggests that the gut microbiota may also influence skeletal muscle health, leading to the emergence of the gut–muscle axis as a novel and rapidly evolving area of research [8,9]. Experimental and observational studies indicate that alterations in microbial composition and function, as well as in microbial-derived metabolites, particularly short-chain fatty acids (SCFAs), may affect muscle metabolism, inflammatory pathways, and anabolic signaling [10,11]. However, whether gut dysbiosis directly contributes to sarcopenia or instead acts as an intermediary between environmental factors, such as diet, physical activity, and medication use, and muscle decline remains a matter of debate [8].

Despite increasing interest in this field, current evidence is largely derived from animal models and observational studies in humans, while interventional data in older adults remain limited. Existing literature has primarily focused on mechanistic pathways or descriptive associations, while the integration of experimental and clinical evidence from a translational perspective remains relatively underexplored [12,13,14,15,16,17,18]. Therefore, this narrative review aims to critically synthesize current mechanistic, clinical, and translational evidence linking the gut microbiota to skeletal muscle health in aging, with particular emphasis on its potential role in the pathophysiology of sarcopenia and on emerging microbiota-targeted interventions.

## 2. Methods

This narrative review is based on a non-systematic literature search conducted in the PubMed database. The search included articles published from database inception through January 2025. The search strategy combined Boolean operators and predefined keywords, including terms related to sarcopenia, gut microbiota, and relevant biological mechanisms (e.g., microbial metabolites and intestinal permeability). The search terms were used in an iterative and flexible manner to capture a broad range of potentially relevant studies. Key references were screened by the authors (S.A.M. and S.C.) to identify studies reporting objective muscle-related outcomes (e.g., muscle strength, muscle mass, or gait speed) and clearly defined sarcopenia phenotypes. Both preclinical (animal and in vitro models) and clinical (human observational and interventional) studies were considered. Preclinical models were prioritized for their capacity to identify molecular and mechanistic pathways linking microbiota alterations to muscle metabolism and anabolic signaling, whereas human studies were evaluated for clinical relevance and epidemiological associations. Articles were excluded if they lacked a clear focus on the gut–muscle axis or addressed secondary muscle wasting not primarily related to aging. Study selection was guided by conceptual relevance to the biological framework of the microbiota–gut–muscle axis rather than by formal quality scoring or statistical pooling, consistent with the integrative nature of a narrative review.

## 3. Sarcopenia: Pathophysiology and Current Therapeutic Strategies

Sarcopenia is an age-related skeletal muscle disorder characterized by progressive declines in muscle strength, mass, and physical performance and is associated with an increased risk of frailty, disability, and adverse clinical outcomes in older adults [1]. International consensus definitions have been developed to standardize its identification across clinical and research settings, generally integrating measures of muscle strength, muscle quantity, and physical performance [19]. Sarcopenia remains an evolving field, with ongoing refinements in its conceptual and operational definitions [20,21].

### 3.1. Molecular and Intrinsic Mechanisms

The pathophysiology of sarcopenia is complex and multifactorial, resulting from the interplay between intrinsic biological processes (e.g., cellular and molecular alterations within skeletal muscle) and extrinsic environmental and clinical factors (e.g., physical inactivity, nutrition, and comorbidities). A central molecular mechanism is anabolic resistance, defined as a reduced sensitivity of skeletal muscle to anabolic stimuli such as dietary amino acids and mechanical loading. This leads to impaired activation of key signaling pathways involved in muscle protein synthesis, most notably the IGF-1–Akt–mTORC1. This defect is further compounded by a reduction in satellite cell number and function, thereby limiting muscle regeneration and repair [4,22]. Additionally, mitochondrial dysfunction contributes to muscle degeneration by reducing oxidative capacity, increasing reactive oxygen species production, and impairing energy metabolism, ultimately favoring myofiber atrophy and apoptosis [23].

### 3.2. Systemic, Endocrine, and Neuromuscular Factors

Beyond intrinsic muscle defects, chronic low-grade systemic inflammation (inflammaging) represents a hallmark of the disorder. Persistently elevated circulating levels of pro-inflammatory cytokines, such as tumor necrosis factor alpha, interleukin 6, and C-reactive protein, promote proteolysis through activation of the ubiquitin–proteasome and autophagy–lysosome systems [22,24]. In parallel, age-related alterations in neuromuscular junction integrity, including motor neuron loss and impaired reinnervation, lead to motor unit remodeling and preferential denervation of fast-twitch fibers, exacerbating strength decline [25]. Furthermore, endocrine changes, characterized by reduced levels of testosterone, estrogen, growth hormone, and vitamin D, negatively affect muscle mass maintenance and neuromuscular function [26].

### 3.3. Therapeutic Landscape

Current therapeutic strategies primarily focus on resistance exercise training and optimization of protein and energy intake, which remain the cornerstone interventions. Resistance exercise counteracts anabolic resistance by stimulating muscle protein synthesis and improving neuromuscular function, while adequate protein intake, particularly from leucine-rich sources, supports anabolic signaling [1,4,27]. However, responses to these interventions are heterogeneous and influenced by age, comorbidities, physical inactivity, and potentially by individual biological and environmental factors, including gut microbiota composition. Importantly, no pharmacologic therapies are currently approved for the treatment of sarcopenia [28].

## 4. Gut–Muscle Axis: Biological and Translational Insights

**Figure 1:**
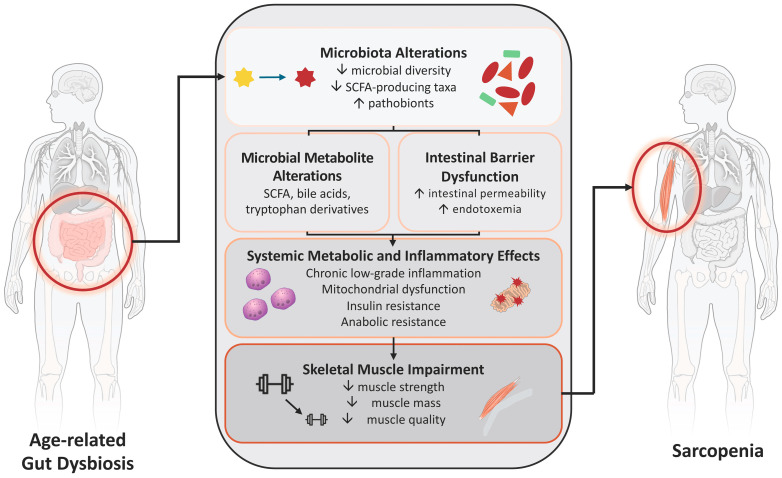
Conceptual framework of the gut–muscle axis in aging. The figure illustrates the interconnected pathways linking age-related gut dysbiosis to skeletal muscle impairment, including microbiota alterations, microbial metabolite changes, intestinal barrier dysfunction, and downstream systemic metabolic and inflammatory effects. Connections represent biologically plausible interactions, including reciprocal relationships between microbiota composition, metabolite production, and intestinal barrier integrity. Abbreviation: SCFA, short-chain fatty acid.

Both preclinical and human studies support a role for the gut microbiota in the regulation of skeletal muscle mass, strength, and function, although the underlying mechanisms remain incompletely understood. The proposed mechanisms linking age-related gut dysbiosis to impaired skeletal muscle health are summarized in Figure 1.

In animal models, across a wide range of experimental settings, including physiological aging, frailty, glucocorticoid-induced muscle atrophy, antibiotic-induced dysbiosis, and fecal microbiota transplantation, converging evidence indicates that alterations in gut microbial composition or microbial-derived metabolites can influence skeletal muscle phenotype. This has been consistently demonstrated through probiotic and postbiotic supplementation studies [29,30,31,32,33,34,35,36,37,38,39,40,41,42,43,44,45], germ-free and colonization experiments [46,47], as well as fecal microbiota transplantation from donors with divergent physical function [48,49,50], collectively showing effects on muscle mass, strength, endurance, and exercise adaptation (Table 1). These effects are mediated through interconnected pathways involving systemic and local inflammation, mitochondrial function, anabolic signaling, and proteostasis, thereby suggesting a potential causal contribution of the gut microbiota to the regulation of muscle mass, strength, and functional performance.

Collectively, preclinical findings support the concept of a gut–muscle axis, whereby gut dysbiosis may contribute to sarcopenia through interconnected pathways, including altered protein metabolism, chronic low-grade inflammation, metabolic resistance, and mitochondrial dysfunction. The following sections summarize the key pathophysiological mechanisms linking gut microbiota alterations to skeletal muscle decline with aging. Notably, the strength of evidence appears to vary across the translational continuum, with robust mechanistic support from preclinical models and consistent observational associations in humans but limited causal inference.

### 4.1. Gut Microbiota Ecosystem Shifts and Skeletal Muscle Homeostasis

Age-related gut dysbiosis is characterized by a significant reduction in microbial diversity and a profound shift in the gut ecosystem [51,52]. This transition involves the depletion of beneficial commensal taxa, such as *Faecalibacterium prausnitzii*, *Roseburia* spp., *Eubacterium rectale*, and *Bifidobacterium* spp., alongside the enrichment of pro-inflammatory pathobionts, including *Enterobacteriaceae* (e.g., *Escherichia coli*), *Desulfovibrio* spp., and *Bilophila wadsworthia* [51,52].

Crucially, sarcopenia is not merely associated with the loss of individual taxa but with broader alterations in relative abundance and microbial ratios that may compromise the stability of the intestinal environment [9,10,16]. In addition to shifts in individual taxa, aging-related dysbiosis has also been described in terms of broader ecological changes at higher taxonomic levels. In particular, an increased relative abundance of *Proteobacteria*, often expressed as the *Proteobacteria*-to-total bacteria ratio, has been proposed as a marker of microbiome instability and inflammatory dysbiosis [52,53]. Conversely, commonly reported indices such as the *Firmicutes*-to-*Bacteroidetes* ratio show inconsistent associations with aging and age-related conditions and are increasingly considered insufficient as standalone indicators of microbiome aging [54,55]. These alterations likely reflect complex host–microbiome interactions rather than simple taxonomic imbalances, and their functional implications may vary depending on microbial gene expression and metabolic activity. Moreover, the directionality of these associations remains difficult to disentangle, as lifestyle, metabolic, and clinical factors accompanying aging may simultaneously influence both gut microbiota composition and skeletal muscle health.

Experimental models further demonstrate that global microbiota disruption through antibiotic-induced depletion impairs muscle adaptation and promotes systemic inflammatory signaling [46,47]. This altered ecosystem contributes to increased intestinal permeability, or “leaky gut”, which facilitates the translocation of microbial components such as lipopolysaccharides into the systemic circulation. This process activates innate immune pathways, particularly toll-like receptor 4, contributing to chronic low-grade inflammation (inflammaging). This persistent inflammatory milieu promotes muscle proteolysis and functional decline, as supported by animal studies showing that restoration of microbial balance and ecological diversity attenuates inflammation and preserves the muscle phenotype [37,38,50].

### 4.2. Microbiota-Derived Metabolites and Skeletal Muscle Homeostasis

Microbiota-derived metabolites constitute a central functional link between gut microbial activity and skeletal muscle physiology. Among these, SCFAs, particularly acetate, propionate, and butyrate, are primarily produced by taxa such as *Faecalibacterium prausnitzii*, *Roseburia* spp., and members of *Clostridium* cluster XIVa. Experimental studies demonstrate that probiotic and postbiotic supplementation increases SCFAs availability, leading to improvements in muscle mass, strength, and endurance in aging and atrophy models [29,31,40,41].

Fecal microbiota transplantation from physically robust or young donors restores microbial metabolite profiles and improves muscle metabolic efficiency and endurance capacity in recipient animals [48,49,50].

Beyond SCFAs, altered microbial metabolism of bile acids and tryptophan-derived metabolites has been implicated in modulating muscle inflammation and energy homeostasis, further supporting a metabolite-mediated gut–muscle communication network [56,57,58].

### 4.3. Gut Microbiota and Mitochondrial Function in Aging Muscle

Mitochondrial dysfunction is a hallmark of skeletal muscle aging and plays a central role in the development of sarcopenia [59]. Emerging experimental evidence indicates that gut dysbiosis may exacerbate age-related mitochondrial impairments through both inflammatory and metabolic pathways. Antibiotic-induced microbiota depletion results in reduced mitochondrial biogenesis, impaired oxidative phosphorylation, and decreased adenosine triphosphate production in skeletal muscle [46,47]. Conversely, supplementation with probiotics such as *Lactobacillus plantarum*, *Lacticaseibacillus rhamnosus*, and *Bifidobacterium* spp., as well as microbial-derived metabolites, improves mitochondrial content, oxidative capacity, and endurance performance in aging and muscle atrophy models [32,36,42].

Fecal microbiota transplantation studies further demonstrate that donor-dependent microbial profiles influence mitochondrial efficiency and exercise tolerance in recipient animals [48,50]. These findings suggest that gut microbiota composition and metabolic output may represent important modulators of mitochondrial health in skeletal muscle.

### 4.4. Protein Anabolism, Amino Acid Metabolism, and Anabolic Resistance

Anabolic resistance, defined as a reduced muscle protein synthetic response to anabolic stimuli [60], is a defining feature of sarcopenia and may be partially mediated by gut microbiota-dependent regulation of amino acid metabolism.

Experimental studies indicate that probiotic supplementation enhances anabolic signaling pathways, including activation of Akt/mTORC1 signaling and increased expression of muscle protein synthesis markers [30,33,43]. Gut microorganisms influence the bioavailability and metabolism of essential amino acids, particularly branched-chain amino acids, which are critical activators of muscle protein synthesis [61,62].

Dysbiosis-induced alterations in microbial composition may impair amino acid absorption and signaling, thereby contributing to anabolic resistance. Consistent with this concept, microbiota depletion models demonstrate impaired anabolic responses and reduced exercise-induced muscle adaptation [46].

Fecal microbiota transplantation and colonization experiments further support a role for microbial composition in shaping host nutrient utilization and muscle anabolic capacity [49,50].

### 4.5. Insulin Resistance and Endocrine Alterations

Insulin resistance frequently accompanies aging and represents an additional pathway linking gut dysbiosis to skeletal muscle decline [63]. Insulin and insulin-like growth factor 1 (IGF-1) signaling are critical for muscle glucose uptake, protein synthesis, and maintenance of muscle mass [63].

Alterations in gut microbiota composition and microbial metabolite production may influence systemic insulin sensitivity through inflammatory signaling, bile acid metabolism, and modulation of incretin pathways. Experimental studies suggest that microbiota-targeted interventions may improve metabolic regulation and physical performance in preclinical models [31,42].

In humans, observational studies associate specific microbial signatures with metabolic profiles relevant to sarcopenia and insulin resistance [64,65]. Reduced SCFAs production and increased endotoxemia may impair insulin and IGF-1 signaling in skeletal muscle, thereby exacerbating anabolic resistance and muscle wasting. Restoration of microbial balance may therefore represent a strategy to support endocrine and metabolic regulation of skeletal muscle during aging.

Importantly, these mechanisms do not operate in isolation. Increased intestinal permeability, altered metabolite production, mitochondrial dysfunction, anabolic resistance, and insulin resistance likely reinforce each other, forming a self-perpetuating network that lowers the threshold for muscle decline in aging.

### 4.6. Evidence from Human Observational Studies

In humans (Table 2), observational studies consistently support an association between gut microbiota alterations and sarcopenia-related traits, although the specific patterns identified vary across populations and clinical settings. In older adults with sarcopenia or pre-sarcopenia, reduced alpha diversity and clear differences in community structure have been reported compared with controls, together with a lower relative abundance of *Firmicutes* and depletion of SCFA-producing genera such as *Roseburia*, *Eubacterium*, and *Lachnospira*, which were positively correlated with appendicular skeletal muscle index and grip strength [64]. By contrast, other studies did not detect major differences in overall alpha or beta diversity, but still identified taxon-specific shifts associated with sarcopenia, including lower abundance of beneficial taxa such as *Prevotella* and *Prevotella copri* (primarily associated with propionate production), *Agathobacter* (a butyrate producer), and *Dorea* (with less well-characterized metabolic profiles) [65,66,67,68,69]. These findings suggest that sarcopenia may be linked either to broad ecological disruption or to more selective compositional changes, depending on the underlying population and phenotype studied.

Additional evidence comes from community-dwelling older women, in whom sarcopenia was associated not only with lower muscle mass, weaker grip strength, and slower gait speed, but also with lower fecal concentrations of total SCFAs and butyrate, reduced microbial richness, and enrichment of potentially unfavorable taxa such as *Bacteroides* and *Shigella*, alongside lower abundance of *Agathobacter* and *Dorea* [70]. In the same study, lower protein and fiber intake was also independently associated with sarcopenia, reinforcing the concept that diet, microbiota-derived metabolites, and muscle health are tightly interconnected [70].

In patients with hematological diseases undergoing hematopoietic stem-cell transplantation, microbiota restructuring over time was accompanied by worsening anthropometric and functional parameters, including reductions in body mass index, calf circumference, and gait speed, while pre-transplant sarcopenia was associated with lower *Dorea* and higher *Phascolarctobacterium* abundance [71]. These observations extend the gut–muscle axis concept beyond community settings to highly vulnerable clinical populations.

Observational data also suggest that microbiota–muscle associations may differ by sex. In a large population-based study, higher skeletal muscle mass index in males, but not females, was associated with greater alpha diversity and higher abundance of taxa including *Haemophilus parainfluenzae*, *Roseburia faecis*, and *Lachnospiraceae*-related species after adjustment for age, body mass index, and physical activity [72]. Beyond these observational findings, sex hormones may contribute to shaping the gut–muscle axis through effects on microbiota composition, inflammatory pathways, and muscle metabolism [73]. Estrogen has been associated with preservation of microbial diversity and anti-inflammatory signaling [74,75,76], whereas age-related declines in testosterone may influence muscle protein metabolism and metabolic regulation [77].

Taken together, these studies indicate that microbiota alterations may accompany poorer muscle health, although the specific microbial signature appears to be context-dependent. Importantly, these findings remain observational and do not establish causality. Overall, the available evidence supports a biologically plausible and potentially bidirectional relationship within the gut–muscle axis, whereby gut microbiota may influence muscle metabolism and function, while muscle activity and exercise can, in turn, modulate microbiota composition and metabolic output, underscoring the need for longitudinal studies with deeper phenotyping and functional microbiome characterization.

## 5. Interventional Evidence and Multimodal Strategies Targeting the Gut–Muscle Axis

In contrast to observational findings, interventional studies in humans (Table 3) provide emerging but still limited evidence on the effects of microbiota-targeted strategies on muscle health. Randomized and non-randomized interventions using prebiotics, probiotics, synbiotics, or fecal microbiota transplantation have reported modest and domain-specific improvements, particularly in muscle strength, lower-limb function, and patient-reported outcomes, rather than consistent changes in muscle mass. However, these studies are characterized by heterogeneous designs, small sample sizes, variable endpoints, and limited mechanistic characterization, which currently preclude firm conclusions regarding efficacy. In particular, prebiotic and probiotic interventions have shown improvements in handgrip strength, gait speed, chair stand performance, and quality of life, often in the absence of significant changes in muscle mass or global frailty indices [78,79,80]. In contrast, microbiota-targeted approaches combined with exercise, such as fecal microbiota transplantation plus resistance training, have demonstrated more consistent effects on both muscle mass and functional outcomes, alongside restoration of beneficial microbial taxa and reduction in inflammatory markers [81]. In the study by Yang et al. [81], resistance training consisted of a structured program performed 2–3 times per week, with sessions lasting at least 30 min over a minimum duration of 8 weeks.

**Figure 2:**
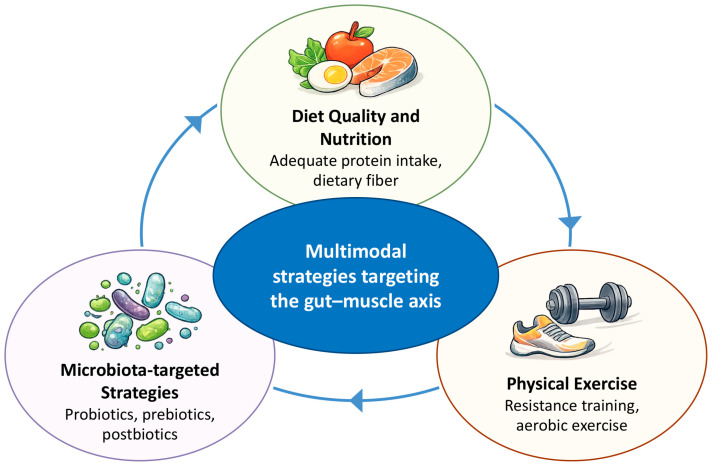
Multimodal interventions targeting the gut–muscle axis. The figure represents a simplified conceptual framework; arrows indicate potential interactions between domains and do not imply fixed or unidirectional causal relationships.

These limitations, including modest and inconsistent effects of microbiota-targeted interventions, small sample sizes, and heterogeneous study designs, have led to increasing interest in integrative and multimodal therapeutic strategies that combine dietary optimization, physical activity, and targeted modulation of the gut microbiota to counteract sarcopenia (Figure 2). Nutritional adequacy, particularly sufficient and sustained protein intake, alongside fiber-rich and high-quality dietary patterns, may support muscle anabolism both directly and indirectly through favorable modulation of gut microbiota composition and metabolic activity [84,85,86]. Dietary fiber promotes the growth of beneficial microbial taxa and enhances SCFAs production, thereby exerting anti-inflammatory effects and supporting anabolic signaling and muscle metabolism [84,85].

Physical exercise, especially resistance training, represents the cornerstone of sarcopenia management and a critical component of multimodal approaches. Resistance training directly stimulates muscle hypertrophy and strength through mechanical loading, activation of anabolic signaling pathways, and myokine secretion [87]. In parallel, regular physical activity influences gut microbiota diversity, composition, and metabolic output, potentially amplifying exercise-induced muscle adaptations through the gut–muscle axis [88]. Exercise-associated increases in microbial diversity and short-chain fatty acid production may further support mitochondrial function, insulin sensitivity, and muscle performance [14].

In addition to protein and fiber intake, other dietary components such as polyphenols and omega-3 fatty acids may contribute to muscle health through antioxidant, anti-inflammatory, and membrane-stabilizing effects, which may synergize with microbiota-mediated mechanisms [17,89]. These nutritional factors are increasingly recognized as modulators of systemic inflammation and microbial metabolic activity, aligning with mechanistic frameworks proposed in integrative reviews [90,91].

Targeted modulation of the gut microbiota represents a promising adjunctive strategy to support musculoskeletal health in aging. This can be achieved through prebiotics, probiotics, and postbiotics, which aim to selectively enhance beneficial microbial populations, increase microbial-derived metabolites, and improve intestinal barrier integrity [86,92]. Prebiotics, such as inulin and fructooligosaccharides, selectively stimulate the growth of taxa including *Bifidobacterium* and *Lactobacillus*, while probiotics consist of live microorganisms that confer health benefits when administered in adequate amounts. Postbiotics, including short-chain fatty acids and other bioactive microbial metabolites, may exert direct biological effects independently of live bacteria. Collectively, these approaches may reduce systemic low-grade inflammation, enhance branched-chain amino acid bioavailability, and support anabolic signaling in skeletal muscle [84,86,93], as highlighted in recent mechanistic and translational syntheses [17].

Importantly, microbiota modulation should be viewed as an amplifier of established interventions such as exercise and adequate protein intake, rather than a replacement.

## 6. Limitations of Current Microbiota-Targeted Strategies and Future Research Directions

Despite growing interest in microbiota-targeted interventions for sarcopenia, several limitations currently hinder their clinical translation. Most available evidence derives from preclinical models, while human interventional studies remain scarce, characterized by small sample sizes, and highly heterogeneous in terms of study design, participant characteristics, intervention duration, and outcome measures [92]. Probiotic and prebiotic trials differ substantially in strains, dosages, formulations, and co-interventions, limiting comparability and precluding firm conclusions regarding efficacy. Notably, available human trials more consistently report improvements in functional outcomes than in muscle mass, suggesting a potential dissociation between functional and structural endpoints, which may partly reflect methodological limitations in muscle mass assessment. Commonly used techniques such as dual-energy X-ray absorptiometry and bioelectrical impedance analysis may lack sensitivity to detect small or short-term changes [94].

Moreover, many studies assess clinical endpoints without adequately characterizing microbiota changes or downstream mechanisms, thereby limiting causal inference. Several trials report clinical improvements without parallel microbiome assessment, while others show microbiota modulation without functional benefits. The lack of standardized analytical methods, limited integration of multi-omics approaches, and insufficient consideration of host factors further complicate interpretation [95]. Most studies rely on taxonomic profiling, which may not fully capture microbial functional capacity; complementary approaches such as metagenomics and metabolomics are needed [96]. Emerging strategies such as fecal microbiota transplantation, although mechanistically informative, raise ethical and feasibility concerns and are supported by limited clinical evidence [97].

Future research should prioritize well-designed, mechanistically informed clinical trials with standardized muscle outcomes and integrated microbiome analyses. Increasing evidence also points to substantial inter-individual heterogeneity in microbiome aging trajectories [98,99], highlighting the need to identify responder subgroups and develop personalized approaches. Integrating microbiome science within multimodal sarcopenia interventions will help clarify the role of microbiota-targeted strategies in prevention and management.

## 7. Conclusions

The emerging concept of a microbiota–gut–muscle axis provides a biologically plausible framework linking gut microbial ecology with skeletal muscle health in aging. However, current evidence remains largely associative, and microbiota modulation should be considered an adjunct to established strategies such as exercise and nutritional optimization rather than a standalone therapeutic approach. Available interventional evidence suggests modest and context-dependent benefits, primarily on functional outcomes, with limited and inconsistent effects on muscle mass. Future research should focus on mechanistically informed clinical studies integrating microbiome profiling with standardized muscle outcomes to clarify causality and identify individuals most likely to benefit from microbiota-targeted interventions.

## Figures and Tables

**Table 1:** Evidence from animal models supporting a causal role of the gut–muscle axis.

Experimental Approach	Experimental Models and Interventions	Muscle-Related Outcomes	Key Mechanisms	References
Probiotic, postbiotic, and microbial metabolite supplementation	Probiotics (*Lactobacillus*, *Bifidobacterium*, *Faecalibacterium*), postbiotics, microbial metabolites	↑ muscle mass ↑ strength ↑ endurance ↓ atrophy	↑ SCFAs ↑ AKT/mTORC1 ↓ inflammation ↑ mitochondrial function	[29,30,31,32,33,34,35,36,37,38,39,40,41,42,43,44,45]
Microbiota depletion and dysbiosis models	Antibiotic-induced dysbiosis	↓ muscle mass ↓ strength ↓ exercise adaptation	↓ anabolic signaling ↓ mitochondrial biogenesis	[46,47]
Germ-free, colonization, and fecal microbiota transplantation models	Germ-free mice, aged microbiota colonization, FMT	Variable effects on strength and endurance (donor-dependent)	↓ SCFAs ↑ inflammation mitochondrial dysfunction	[48,49,50]

↑ indicates an increase or upregulation, ↓ indicates a decrease or downregulation. Abbreviations: AKT, protein kinase B; FMT, fecal microbiota transplantation; mTORC1, mechanistic target of rapamycin complex 1; SCFA, short-chain fatty acids.

**Table 2:** Observational studies on gut–muscle axis in humans.

Author (Year)	Population; Design	Microbiota-Related Exposure	Muscle Outcomes	Key Findings
Kang et al. (2021) [64]	Older adults; case–control (sarcopenia and pre-sarcopenia vs. controls)	Gut microbiota composition	↓ ASMI, ↓ grip strength, ↑ chair stand time (all *p* < 0.05)	Sarcopenia vs. controls: ↓ alpha diversity (*p* < 0.05), distinct beta diversity (R = 0.370, *p* = 0.0001); ↓ Firmicutes; depletion of SCFA-producing genera (*Roseburia*, *Eubacterium*, *Lachnospira*) positively associated with muscle parameters; ↑ LPS pathways
Lee et al. (2022) [65]	Older adults; cross-sectional (sarcopenia vs. controls)	Gut microbiota composition	Sarcopenia defined by ASMI, grip strength, gait speed	Sarcopenia vs. controls: no alpha/beta diversity differences; taxonomic shifts: ↓ *Prevotella*, ↓ *P. copri*, ↑ *Parabacteroides*; positive associations with sarcopenia: *Anaerotruncus*, *Phascolarctobacterium*; negative associations with sarcopenia: *Prevotella*
Yan et al. (2023) [70]	Community-dwelling older women; cross-sectional	Gut microbiota composition and fecal SCFAs	Sarcopenia vs. non-sarcopenia: ↓ ASMI (5.39 vs. 6.62 kg/m^2^), ↓ grip strength, ↓ gait speed (all *p* < 0.001)	Sarcopenia vs. non-sarcopenia: ↓ total SCFAs and butyrate; ↓ richness; ↑ *Bacteroides*, *Shigella*; ↓ *Agathobacter*, *Dorea*; lower protein/fiber intake associated with sarcopenia
Wang et al. (2023) [71]	Patients with hematological diseases undergoing HSCT; observational study with repeated time points	Gut microbiota composition before and after HSCT	Post-HSCT: ↑ severe sarcopenia (3.5% vs. 15–26%); ↓ BMI, ↓ calf circumference, ↓ gait speed	Sarcopenia and progression over time: microbiota restructuring (↑ *Enterococcus*, ↓ *Bacteroides*); pre-HSCT sarcopenia: ↓ *Dorea*, ↑ *Phascolarctobacterium*; beta diversity changes over time (R^2^ = 0.078, *p* = 0.001)
Park et al. (2022) [72]	Adults; population-based cross-sectional study, sex-stratified analysis	Gut microbiota composition across sex-specific quartiles of SMI	Higher SMI (Q4 vs. Q1): males 46.9 vs. 40.4%; females 42.0 vs. 34.3%	Higher muscle mass (males only): ↑ alpha diversity and ↑ *H. parainfluenzae*, *R. faecis, Lachnospiraceae* taxa; no consistent associations in females

↑ indicates increase; ↓ indicates decrease. Abbreviations: ASMI, appendicular skeletal muscle index; BMI, body mass index; HSCT, hematopoietic stem-cell transplantation; SCFAs, short-chain fatty acids; SMI, skeletal muscle mass index.

**Table 3:** Interventional studies targeting the gut–muscle axis in humans.

Author (Year)	Population; Design	Intervention	Duration	Muscle Outcomes	Microbiota Outcomes	Main Findings
Buigues et al. (2016) [78]	Frail older adults living in nursing homes; RCT	Prebiotic formulation (inulin + fructooligosaccharides) vs. placebo (maltodextrin)	13 weeks	↑ handgrip (10.6 vs. 12.4 kg); ↓ exhaustion; no change in walking speed or Barthel index	Not assessed	Improved strength and fatigue, no effect on overall frailty
Qaisar et al. (2024) [79]	Sarcopenic older men; RCT	Multistrain probiotic (Vivomix 112 billion) vs. placebo	16 weeks	↑ handgrip and gait speed (*p* < 0.05); no effect on SMI	↓ plasma zonulin (*p* < 0.05)	Improved function and quality of life without changes in muscle mass
Kang et al. (2024) [82]	Older adults; RCT	Pasteurized *Akkermansia muciniphila* HB05	12 weeks	↑ lower-limb strength (peak torque *p* ≈ 0.01–0.05); no change in grip strength	Not assessed	Improved muscle strength and ↑ follistatin; no effect on myostatin
Ni Lochlainn et al. (2024) [83]	Older adults (twin pairs); RCT	Prebiotic (inulin) vs. placebo	12 weeks	No effect on chair rise, grip strength, or performance	↑ Bifidobacterium, Actinobacteria; no diversity changes	Microbiota modified without functional improvement
Yang et al. (2025) [81]	Older adults with sarcopenia; retrospective comparative study	FMT + RT vs. RT alone	24 weeks follow-up	↑ ASMI, ↑ strength, ↑ gait speed; higher response rate (47.1% vs. 32.2%)	↑ diversity; ↑ SCFA-producing taxa (*Dorea*, *Roseburia*, etc.); ↓ inflammation	Combined FMT + RT superior to RT alone for clinical and microbiota outcomes
Lee et al. (2025) [80]	Older adults; RCT	*Lacticaseibacillus paracasei* PS23 vs. heat-treated PS23 vs. placebo	12 weeks	No change in grip strength; ↑ chair stand and TUG (*p* < 0.05)	Not assessed	Improved lower-limb function and anti-inflammatory profile

↑ indicates increase; ↓ indicates decrease. Abbreviations: ASMI, appendicular skeletal muscle index; FMT, fecal microbiota transplantation; RT, resistance training; SCFA, short-chain fatty acid; SMI, skeletal muscle mass index; TUG, timed up-and-go test.

## Data Availability

No new data were created or analyzed in this study. Data sharing is not applicable to this article.
